# The Role of Lipid Composition in the Sensory Attributes and Acceptability of the Salted and Dried Mullet Roes (Bottarga): A Study in Human and Animal Models

**DOI:** 10.3390/nu12113454

**Published:** 2020-11-11

**Authors:** Antonella Rosa, Raffaella Isola, Mariella Nieddu, Carla Masala

**Affiliations:** Department of Biomedical Sciences, University of Cagliari, Cittadella Universitaria, SS 554, km 4.5, Monserrato, 09042 Cagliari, Italy; isola@unica.it (R.I.); mnieddu@unica.it (M.N.); cmasala@unica.it (C.M.)

**Keywords:** mullet roes, rich-fat food, lipids, free fatty acids, flavor, odor, taste

## Abstract

A taste component is implicated in the oro-sensory detection of dietary lipids and free fatty acids seem to be involved in fatty food recognition. Bottarga, the salted and semi-dried ovary product of mullet (*Mugil* spp.), is a rich-fat food. A comparative sensory assessment of different commercial bottarga samples was performed in insect and human models in relation to their lipid composition. The bottarga attractant effect to *Ceratitis capitata* was assessed by behavioral tests. The subjective odor and taste perception of bottarga samples was investigated in human determining the rate of pleasantness, familiarity, and intensity dimensions using the 7-points Likert-type scale. Bottarga samples showed similar lipid profiles, but differences emerged in total and free fatty acid levels. Significant differences were observed in the attractant effect/acceptability of samples to medflies, negatively correlated to their total and free fatty acids. Insect female exhibited the ability to select among bottarga samples based on their visual and olfactory properties. In the human model, a potential contribution of free fatty acid amount in the pleasantness and familiarity dimensions of taste of bottarga samples was evidenced. Women exhibited a greater ability than men to select bottarga samples based on their better olfactory perception. Our results increase the knowledge about this outstanding product with nutritional and nutraceutical properties.

## 1. Introduction

Female fish roes (the egg-laden ovary) are highly valued gourmet products, generally consumed raw, frozen, or as salted, boiled, canned, and smoked products [1,2,3]. The salted and semi-dried mullet (*Mugil* spp.) ovary product is produced in numerous world countries. This food delicacy is commonly known with the name of “bottarga” (Italy) [4,5], “avgotaracho” (Greece) [2] and ‘‘karasumi” (Japan) [1]. The island of Sardinia (Italy) has a long tradition in manufacturing a bottarga of high quality, also exported around the world [5]. Bottarga is a “Traditional product of Sardinia”, thus the roes used for its manufacturing can be of local origin or imported as frozen roes, then processed by Sardinian companies according to tradition [6,7,8]. During the industrial production, eviscerated mullet roes (intact ovaries) are washed with water and salted with sea salt. After excess salt removal, roes were then pressed and dried in rooms with controlled conditions of temperature and humidity (cured roes) [1,5,6]. Bottarga is generally sold as whole ovaries (undamaged ovarian sacs) under vacuum packaging or grated (in jars or bags) [5,9]. We have previously studied the chemical composition [5,8,9,10], stability to the oxidative degradation [9,10,11], non-enzymatic browning process [9,10,11], biological profile in normal and cancer cells [11,12,13], and bioavailability in cell systems and rat model [11,12,13,14] of Sardinian bottarga samples. Bottarga is a stable natural source of health beneficial *n*-3 polyunsaturated fatty acids (*n*-3 PUFA or *ω*-3 PUFA) (13–25% of total fatty acids) [5,9,10,11], especially eicosapentaenoic acid (EPA, 20:5 *n*-3) and docosahexaenoic acid (DHA, 22:6 *n*-3), mostly in the form of wax esters (about 50–65% of total lipids) [5,10,13,15]. It is considered a highly nutritive food for its richness in vitamins and well-balanced proteins with essential amino acids [1,2]. We also evidenced the presence of a high content of free fatty acids (FFA) and PUFA degradation products in commercial whole and grated bottarga samples, due to hydrolysis and oxidation processes induced on the raw matrix by the manufacturing/storage procedures and conditions [5,9,10,11]. Therefore, bottarga physico-chemical characteristics, quality, and sensory properties may change according to the provenience and quality of raw materials, differences in manufacturing and storage conditions that can affect hydrolytic and degradative processes on lipid and protein components [5,9,10,11].

The concept of food quality and the relation among food quality, sensory attributes and consumer acceptance have received a lot of attention during the last 30 years [16,17,18]. Food intake control is greatly influenced by the sensory experience of eating [18] and sensory quality is considered a key factor in food acceptance [17]. Appearance, texture, aroma, taste, and irritation, that represent the five major sensory properties of food materials, are perceived by primary human senses [19] and sensory cues are operational in all phases of an eating event [18]. “Flavor”, an attribute of foods, is perceived through a combination of olfactory and gustatory information [20]. In facts, a percentage of 80–90% of the food taste quality arrives from the nose [20]. In particular, the flavor of a specific food is carried out by trigeminal and glossopharyngeal nerves together with retronasal olfaction. The specific common pathways between olfactory and gustatory information is well known in animal models [21]. The five basic taste modalities commonly recognized are sour, salty, sweet, bitter, and umami (the taste of mono sodium glutamate) [22]. Olfactory and gustatory information usually converges in the orbitofrontal cortex and in the anterior insula [23]. The texture and fat content are important factors that influence food palatability [23]. Rodents and humans share an attraction for palatable fat-rich foods [22,24,25] and a taste component has been implicated in the oro-sensory detection of dietary lipids (especially long-chain fatty acids) in addition to olfactory and textural cues [22]. Furthermore, FFA seem to be involved in fatty food recognition [24,26].

In view of the fact that the traditional food product bottarga is a palatable food rich in lipids [22] and factors such as manufacturing, processing, or storage methods can impact its characteristics and acceptability [27], the purpose of the present study was to perform a comparative sensory assessment of different commercial bottarga samples in relation to their lipid composition. We investigated for the first-time differences in the sensory properties (taste and odor) and acceptability of three different grated bottarga samples in order to assess whether those differences were associated with their total FA (TFA) and FFA composition. Main lipid classes, TFA and FFA levels were analyzed in bottarga samples by high performance liquid chromatography (HPLC). Bottarga sensory properties, food acceptance and feeding behavior were assessed in insect (*Ceratitis capitata*) and human models. Medfly possesses an extremely sensitive and selective olfactory system, and its general acceptance of extracts/foods can be evaluated using choice assays [28]. The subjective odor and taste perception of bottarga samples was investigated in human determining the rate of three different dimensions: pleasantness, familiarity and intensity [29], using the 7-points Likert-type hedonic scale. A better understanding of the oro-sensory properties of commercial bottarga samples and consumer acceptability is expected to aid the development of this traditional food product with nutritional and nutraceutical properties.

## 2. Materials and Methods

### 2.1. Chemicals

Standards of FA, cholesterol, triacyclglycerols (TAG), cholesteryl esters (CE), phospholipids (PL), wax esters (WE), and high purity solvents were obtained from Sigma-Aldrich (Milan, Italy). cis, trans-13-Hydroperoxyoctadecadienoic acid and cis, trans-9-hydroperoxyoctadecadienoic acid were purchased from Cascade Biochem Ltd. (London, United Kingdom). The yeast used for insect diet was obtained from Il Fior di loto Srl (Torino, Italy). The ultrapure water was obtained by distillation and filtration through the Milli-Q apparatus (Millipore, Milan, Italy). All chemicals used were of analytical grade.

### 2.2. Bottarga Samples

Commercial samples of mullet grated bottarga (bottarga 1 (B1), bottarga 2 (B2), bottarga 3 (B3)) were produced by local manufacturers (Mediterranea Conserve Alimentari S.r.l. and Sud Ovest Bottarga, Sardinia, Italy) according to the Sardinian traditional practices. The main ingredients reported on the labels were: mullet roes and salt. Mullets were caught in the fishing area FAO (Food and Agriculture Organization) 34 for all bottarga samples. The drying procedure of B1 was conducted until 45–50% of the original product weight was removed, while dryness was less marked for products B2 and B3 (35–40%) as specifically indicated by the respective producers. Grinding of product B1 was performed by a Roboqbo cutter (Roboqbo Srl, Bentivoglio, BO, Italy), while a hammer grinder mill was used for the preparation of B2 and B3 samples. Bottarga samples were kept at −60 °C after preparation until analysis.

### 2.3. Microscopic Analysis of Bottarga Samples

Grated bottarga samples were observed using a common digital camera, a binocular and conventional light microscopy to evaluate differences in the color, morphology, and homogeneity of particles. Specimens were simply spread over a microscopy slide and observed with a Leica MS5 binocular microscope (Leica Microsystems Srl, Buccinasco, Italy) equipped with a regular camera or with a Leica DMR-HC light microscope (Leica Microsystems Srl, Buccinasco, Italy) equipped with a Charge-Coupled Device (CCD) color camera. This evaluation was performed to obtain a qualitative estimation of the granulometry of the three different bottarga samples, due to the different drying and grinding procedures.

### 2.4. Extraction and Saponification of Lipids

The chloroform/methanol/water (CHCl_3_/MeOH/H_2_O) mixture (in a ratio 2:1:1) was used for the extraction of total lipids from aliquots (40 mg) of grated bottarga samples (B1, B2 ad B3) as previously reported [9]. A dried aliquot of the CHCl_3_ fractions from each bottarga sample was dissolved in methanol and injected into the high-performance liquid chromatograph (HPLC) system for the qualitative analysis of lipid components, while another dried aliquot, dissolved in ethanol, was subjected to mild saponification according to literature [9,12]. The unsaponifiable and saponifiable fractions were separated and total cholesterol (TC), total FA, and conjugated diene fatty acid hydroperoxides (HP) were analyzed by HPLC system [9,12].

### 2.5. Analysis of Lipid Components

The analyses of lipid compounds of bottarga samples were performed with a 1100 HPLC (Agilent Technologies, Palo Alto, CA, USA) equipped with a diode array detector (DAD) and a 1260 infinity evaporative light scattering detector (ELSD) (Agilent Technologies, Palo Alto, CA, USA) [13]. The qualitative profile of FFA, free fatty alcohols (FFAl), wax esters (WE), phospholipids (PL), triacyclglycerols (TAG), and free cholesterol (FC) in bottarga lipid extracts [13] was determined with an Inertsil ODS-2 column (Superchrom, Milan, Italy), methanol (at a flow rate of 2 mL/min) as mobile phase, and ELSD detection. The ELSD operating parameters: 40 °C was the evaporator and nebulization temperature; nitrogen (at a flow of 1 L/min) was the nebulizing gas. The total cholesterol (TC) quantification after saponification was conducted with the same column, DAD detection (at wavelength of 203 nanometers (nm)), and methanol as mobile phase (flow rate of 0.7 mL/min) [9,13]. After saponification, the analyses of unsaturated FA and hydroperoxides (HP) (detected with DAD at wavelengths of 200 and 234 nm, respectively) and saturated FA (ELSD detection) were carried out with a XDB-C_18_ Eclipse column (Agilent Technologies, Palo Alto, CA, USA), using as mobile phase the mixture acetonitrile/water/acetic acid (CH_3_CN/H_2_O/CH_3_COOH) (75/25/0.12, v/v/v) at 2.3 mL/min flow rate, as previously reported [13]. The same analytical condition was employed for the FFA quantification in dried chloroform fractions, dissolved in methanol, obtained from lipid extraction of bottarga samples. The Agilent OpenLAB Chromatography data system (Agilent Technologies, Palo Alto, CA, USA) was used for the recording and integration of the chromatogram data. Standards and the conventional and second derivative UV spectra were used for the identification of lipid compounds. The quantification of lipid compounds was based on calibration curves constructed using standards (correlation coefficients >0.995). Calibration curves, in the amount range of 100–1000 ng (nanograms), were linear for unsaturated FA (DAD detection) and quadratic for saturated FA (ELSD detection) [13].

### 2.6. Insects

The population of Mediterranean fruit fly (*Ceratitis capitata*) used for experiments was obtained from a laboratory colony reared in the Department of Biomedical Sciences, University of Cagliari (Italy). The temperature of 22 ± 1 °C and a relative humidity of 70–80% were kept constant throughout all the experiments. Adults of medflies (approximately 100 insects, 1:1 males and females) were kept in cages (dimensions of 47.5 × 47.5 × 47.5 cm) provided with standard diet (represented by a solid mixture of sucrose and yeast 4:1) and water ad libitum as previously reported [28].

### 2.7. Behavioral Tests on Medfly

Experiments were conducted using sexually adult medflies 3 to 5 days old. Flies were deprived of food for 24 h but had free access to water before behavioral tests. In the double-choice behavioral tests, *Ceratitis capitata* could select between the control (pure deionized water or standard diet) and each bottarga sample (B1, B2 or B3). In the initial set of experiments, medfly adults of both sexes (about 60 in each cage) had simultaneously free access to grated bottarga samples (3 g) compared to water (3 mL), both presented on a filter paper disk positioned on a Petri dish [28]. Whereas, in the second set of double-choice experiments, medflies had free access to each bottarga sample, compared to the standard diet (control). A third set of experiments was carried out to record the medfly capacity to choose among the three bottarga samples (multiple-choice behavioral test). Bottarga attractant effect in all behavioral tests was evaluated by counting the number of insects that was attracted in 1 h to each Petri dish and got in touch with tested samples through tarsal and labellar apparatus [28]. In the two-choice test, the attractiveness was expressed as the relative percentage of visits in relation to the total number of insects that entered in contact with the control and each bottarga sample. In the multiple-choice test, the attractiveness was determined as the relative % of visits to each bottarga sample with respect to the total number of insects that entered in contact with the three bottarga samples. During the experimental sets, the % number of insect males and females attracted to each Petri dish was also determined.

### 2.8. Participants

Fifty-nine participants were enrolled in this study, 24 men and 35 women, with an age range 19–64 years (mean age of 37.3 ± 14.6). All participants received an explanatory statement and furnished their written informed consent to participate in the research study. Exclusion criteria were the absence of neurodegenerative diseases, chronic/acute rhinosinusitis, and systemic smell disorders as reported in previous studies [30,31]. None of the participants was taking medications (for allergies or other diseases) for 5 days before the test. These conditions were checked by the examiner closely before the starting of procedure.

### 2.9. Procedures to Assess Pleasantness, Intensity and Familiarity in Odor and Taste for Bottarga Samples in a Human Model

In human model pleasantness, intensity and familiarity for odor and taste of the three different mullet bottarga samples were assessed using the 7-points Likert-type scale [30]. The Likert-type scale usually ranges from one extreme to another, for example “very intense” to “not intense at all”. In order to analyze and interpret the Likert Scale with 7 points, a point value was assigned for each response ranging from 0–6 (such as 0 = very unpleasant and 6 = very pleasant; 0 = not intense at all and 6 = very intense; 0 = not familiar at all and 6 = very familiar). The order of odor and taste stimuli was randomized. Participants were asked to evaluate the rate of pleasantness, intensity and familiarity of the odor and taste qualities in the different grated bottarga samples (B1, B2 and B3). All participants could drink only water 1 h before the experiment and not wear any perfumed products during the day of testing [31,32]. This study was approved by the local Ethics Committee (Prot. PG/2018/10157) and was performed according to the Declaration of Helsinki.

### 2.10. Statistical Analyses

Graph Pad INSTAT software v3.0 (GraphPad software, San Diego, CA, USA) was used to evaluate statistical differences. Comparison between data groups was assessed by the one-way analysis of variance (ANOVA) followed by the Bonferroni Multiple Comparisons Test (post hoc test) and the Student’s unpaired *t*-test with Welch’s correction. The values with *p* < 0.05 were regarded as statistically significant. The correlation coefficients between sets of data determined for the three bottarga samples were calculated to examine the relationships.

## 3. Results

### 3.1. Visual and Microscopic Analysis

**Figure 1:**
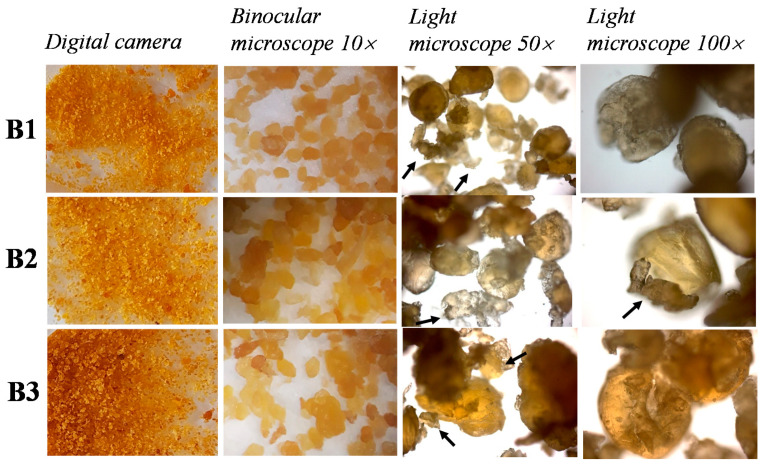
Images of the three grated bottarga samples (bottarga 1 (**B1**); bottarga 2 (**B2**); bottarga 3 (**B3**)). The first column of images shows digital camera photos taken on samples, where the typical color of the samples and the grinding fine sizes can be appreciated. The second column shows binocular microscope images, where it is possible to observe small differences in granulometry. The third and fourth columns are micrograph taken at light microscopy at two different magnifications. As you can see, besides the mullet eggs (better visible at higher magnification), lots of debris are a part of the grinded bottarga (arrows).

Visual observation of B1 and B2 samples revealed a golden yellow color in both (Figure 1).

Observation of B3 sample showed a browning process more marked than in B1 and B2. Microscopic observation (Figure 1) evidenced differences in dimensions of granules of grated bottarga samples. B1 particles were characterized by the smallest size and they were very similar one another, resulting in the most homogeneous grated sample. B2 sample was similar to B1 one, both in the color and in the quality of the particles, which were slightly bigger than those of B1 and a little less homogeneous. B3 specimen had a very variable size of the particles that could be smaller or much huger than the average in the other two samples. In all samples, at a bigger magnification, eggs of mullet were discernible (Figure 1, last column), but also a wide variety of debris were part of the grated bottarga (Figure 1 arrows). The latter were probably remnants of the ovary skin and parts of broken eggs.

### 3.2. Main Lipid Components

**Figure 2:**
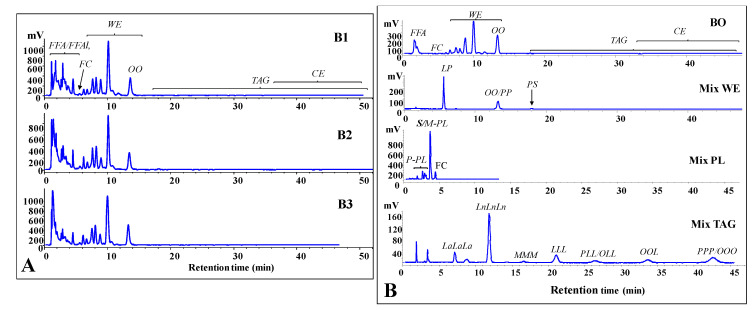
Chromatographic profile (by HPLC-ELSD analysis) of lipids extracted from bottarga samples (B1, B2 and B3) (**A**). Standard mixtures were used for the assignment of the chromatographic region (**B**) of each bottarga lipid class: wax esters (Mix WE: oleyl oleate, OO; palmityl palmitate, PP; palmityl stearate, PS; linoleyl palmitate, LP); saturated, monounsaturated and polyunsaturated phosphatidylcholines (Mix PL: 16:0/16:0, 18:1/18:1, 16:0/18:1, 18:1/16:0, 16:0/18:2, 16:0/20:4, 18:2/18:2, 20:5/20:5); triacylglycerols (Mix TAG: trilaurin, LaLaLa; trilinolenin, LnLnLn; trimyristin, MMM; trilinolein, LLL; 1,2-dilinoleoyl-3-palmitoyl-rac-glycerol, PLL; 1,2-dilinoleoyl-3-oleoyl-rac-glycerol, OLL; 1,2-dioleoyl-3-linoleoy-rac-glycerol, OOL; tripalmitin, PPP; triolein, OOO). Other abbreviations: free fatty acids (FFA), free fatty alcohols (FFAl), free cholesterol (FC), oleyl oleate (OA), polyunsaturated phospholipids (P-PL), saturated/monounsaturated phospholipids (S/M-PL). The chromatographic profile of SFE bottarga oil (BO) [13] is reported for comparison.

The qualitative profile of lipid classes in bottarga samples was assessed by a reversed-phase liquid chromatography with DAD and ELSD detection (HPLC-DAD/ELSD) on a single run. Figure 2A shows the chromatographic profiles of commercial bottarga samples (B1, B2 and B3). Standard mixtures were used for the assignment of the chromatographic region of bottarga lipid classes (FFA, FC, WE, TAG, CE) (Figure 2B). Figure 2B also shows, for comparison, the lipid chromatographic regions of the oil previously obtained by supercritical fluid CO_2_ extraction from grated bottarga (BO) and analyzed with the same chromatographic conditions [13]. Under the chromatographic conditions employed, polar lipids of bottarga oil (FFAl, FFA, FC, and PL) were separated from neural lipids (WE, TAG, and CE), characterized by higher retention times.

**Figure 3:**
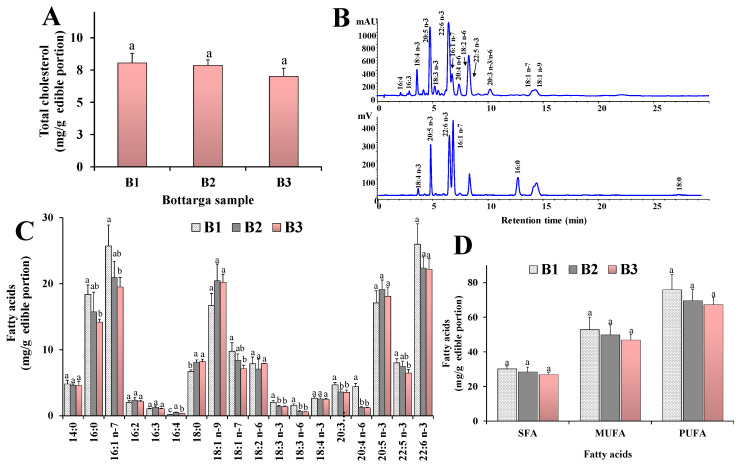
Representative chromatographic profile of fatty acids (FA) of bottarga sample B1 obtained by HPLC analysis with DAD (λ = 200 nm) and ELSD detection (**A**). Values (mg/g of edible portion) of total cholesterol (**B**), main FA (**C**), total saturated (SFA), monounsaturated (MUFA) and polyunsaturated (PUFA) FA (**D**) measured in bottarga samples (B1, B2, B3). The analysis of bottarga oil was performed in quadruplicate and all data are expressed as mean values ± standard deviations (SD); (*n* = 4). Statistically significant differences between bottarga samples B1, B2, and B3 for each data set were determined by One-way ANOVA followed by the Bonferroni Multiple Comparisons Test. Mean values sharing the same superscript letters (a, b, c) are not significantly different from each other, while mean values that have no superscript letters in common are significantly different (level of significance: *p* < 0.05).

The separation of lipid compounds was based on the equivalent carbon number (ECN) = CN - 2*n*, where “CN” indicates the number of acyl group carbons and “*n*” the number of double bonds. Lipids containing fatty acids characterized by the same ECN co-eluted [13,28]. The chromatographic profiles of B1, B2 and B3 were similar and, like BO, were characterized by a high level of peaks in the WE region (main lipid components) and extremely low level of TAG and CE. Moreover, lipid extracts from all bottarga samples were characterized by many peaks in the FFAl and FFA region. The TC level was measured by HPLC-DAD as mean content of 8.04 ± 0.76, 7.85 ± 0.43 and 7.00 ± 0.64 mg/g of edible portion for B1, B2 and B3, respectively (Figure 3A).

### 3.3. Fatty Acid Profile

The FA quali-quantitative profile of lipid classes of bottarga samples was assessed by reversed-phase HPLC-DAD/ELSD analysis. The representative FA chromatographic profile of the bottarga sample B1 obtained by HPLC analysis with DAD (λ = 200 nm) and ELSD detection is shown in Figure 3B. Values of the main saturated (SFA), monounsaturated (MUFA) and polyunsaturated (PUFA) FA measured in bottarga samples (B1, B2 and B3) are shown in Table 1 (expressed as % of total FA) and Figure 3C,D (expressed as mg/g of edible portion).

Bottarga samples showed similar % FA profiles, characterized by SFA (19% of total FA), mostly palmitic acid 16:0 (from 10–12%) and stearic acid 14:0 (4–6%), MUFA (approximately 33%), mainly palmitoleic acid 16:1 *n*-7 (14–16%) and oleic acid 18:1 *n*-9 (10–14%), and PUFA (47–48%), mainly constituted by the highly unsaturated *n*-3 FA, in particular DHA (15–16%) and EPA (10–13%). Among PUFA, bottarga samples were also characterized by a minor amount of 22:5 *n*-3 (4–5%) and linoleic acid 18:2 *n*-6 (approximately 5%). B1 showed a TFA mean content of 158.87 ± 17.40 mg/g of edible portion, while values of 147.67 ± 15.33 and 141.67 ± 8.41 mg/g of edible portion were measured in B2 and B3, respectively. B1 showed concentrations of 30.13 ± 1.83 mg/g of SFA, 52.87 ± 7.10 mg/g of MUFA, and 75.87 ± 8.75 mg/g of PUFA, while *n*-3 PUFA accounted for 55.71 ± 5.89 mg/g of edible portion. B2 and B3 samples showed absolute amounts of SFA, MUFA and PUFA lower (but not in a significant way) than B1 (Figure 3D). Significant differences among bottarga samples were observed in the absolute values of each individual FA, as shown in Figure 3C. The highest absolute FA concentration of B1 sample was due to its lower moisture content with respect to B2 and B3, according to the manufacturing procedures as indicated by the producer. The beneficial EPA and DHA amounted to 26–28% of total fatty acids in B1, B2 and B3. The FFA level, due to the hydrolytic process induced by the manufacturing procedures, was also assessed in bottarga samples. It was calculated that c.a. 29% of FFA (45.70 ± 4.45 mg/g of edible portion) were present in their free form in B1. FFA values of 11% and 13% were respectively found for B2 and B3 samples. No marked differenced were observed in the hydroperoxides HP levels, and HP concentrations in bottarga samples were 1.00 ± 0.03, 0.80 ± 0.01 and 0.65 ± 0.01 μmol/g of edible portion in B1, B2 and B3, respectively.

### 3.4. Behavioral Tests in Insects

**Figure 4:**
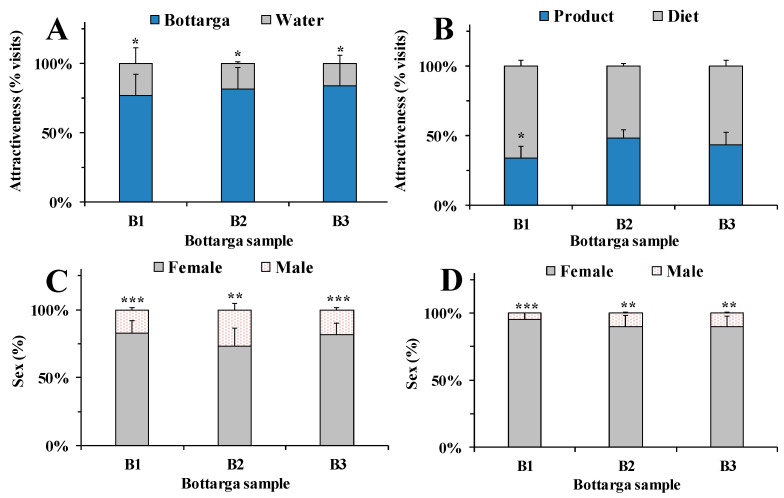
Attractiveness, evaluated by two-choice behavioral tests, of bottarga samples B1, B2 and B3 to *Ceratitis. capitata* determined versus water (**A**) or diet (**B**). Bars represent the relative percentage of visits in relation to the total number of insects that entered in contact with the bottarga sample and the respective control (water/diet). The % values of male and female medflies that entered in contact with each bottarga sample during the two-choice behavioral tests versus water (**C**) or diet (**D**). Six independent experiments are performed, and data are presented as mean ± standard error of the mean (SEM) (*n* = 6). * *p* < 0.05 for bottarga samples vs control (water/diet); *** *p* < 0.001, ** *p* < 0.01 for female vs male medflies (Student’s unpaired *t*-test with Welch’s correction).

Preliminary evaluation of the differences in sensory properties of commercial bottarga samples was assessed in an animal model (*Ceratitis capitata*). The attractant effect of B1, B2 and B3 samples to medflies was assessed by double and multiple-choice behavioral tests. Figure 4A shows the attractant effect of bottarga samples to medflies versus water (control), determined by double-choice tests.

**Figure 5:**
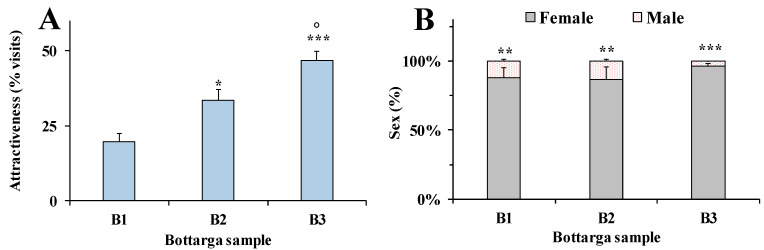
(**A**) Attractiveness, determined by multiple-choice behavioral tests, of bottarga samples (bottarga 1 (B1), bottarga 2 (B2), bottarga 3 (B3)) to medflies. Bars represent the relative percentage of visits to each bottarga sample in relation to the total number of insects that entered in contact with the three bottarga samples. Eight independent experiments are performed, and data are presented as mean ± standard error of the mean (SEM) (*n* = 8). *** *p* < 0.001, * *p* < 0.05 vs B1, ° *p* < 0.05 vs. B2 (One-way ANOVA followed by the Bonferroni Multiple Comparisons Test). (**B**) The % values of male and female medflies that entered in contact with each bottarga sample during the multiple-choice behavioral tests. Data are presented as mean ± standard error of the mean (SEM) (*n* = 8). *** *p* < 0.001, ** *p* < 0.01 for female vs male medflies (Student’s unpaired *t*-test with Welch’s correction).

The attractiveness was expressed as the relative percentage of visits (bottarga sample versus water) with respect to the total number of insects that entered in contact with each bottarga sample and the corresponding control. In this behavioral bioassay, all bottarga samples showed a significant higher attractant effect to medfly with respect to water. The order of attractiveness to insects was: B3 (84% of attractiveness, *p* < 0.05 versus water) >B2 (82%, *p* < 0.05) >B1 (77%, *p* < 0.05). In the double-choice tests versus standard diet (control) (Figure 4B), the number of insects that entered in contact with B2 or B3 (49 and 44% of attractiveness, respectively) and diet was quite close, whereas B1 (33% of attractiveness) showed a less attractant effect than standard diet (*p* < 0.05). The % values of male and female medflies that entered in contact with each bottarga sample during the two-choice behavioral tests versus water (Figure 4C) or diet (Figure 4D) were also determined. Interestingly, in the double-choice tests versus standard diet, a higher value of females (90–95%) was attracted to bottarga samples than males (Figure 4C), while the % ratio between males and females that enter in contact with water in this test was 57:43. During the two-choice tests versus diet (Figure 4D), a marked prevalence of female insects (90–95%) that entered in contact with bottarga samples was also observed while the % ratio of females to males for diet was 78:22. Figure 5 shows the attractiveness (Figure 5A) and the medflies sex ratio (Figure 5B) of bottarga samples B1, B2 and B3 to *Ceratitis capitata* determined during the multiple-choice behavioral tests. The attractant effect was evaluated as the relative percentage of visits to each bottarga sample with respect to the total number of insects that got in touch with the three bottarga samples. Significant marked differences were observed in the attractant effect of bottarga samples to medflies, and the order of attractiveness was B3 (47%) > B2 (33%) > B1 (20%). In this test, the % of insect females that entered in contact with bottarga samples B1, B2 and B3 was in the range 87–96%, with the highest value observed for B3. The correlation between bottarga lipid content and insect attractiveness was determined with the intention to understand the role of fat in the medfly feeding behavior (Table 2).

The bottarga attractant effect on insects was negatively correlated to its concentration of TFA and FFA, as indicated by the high negative correlation coefficients (r) between bottarga sample attractiveness/TFA (r = −0.9909) and attractiveness/FFA (r = −0.8426), moreover similar correlation was also found with SFA (r = −0.9996), MUFA (r = −1.0000) and PUFA values (r = −0.9663) (Table 2). B1 sample, characterized by the highest TFA and FFA amount, was less attractant than B2 and B3.

### 3.5. Human Model

**Figure 6:**
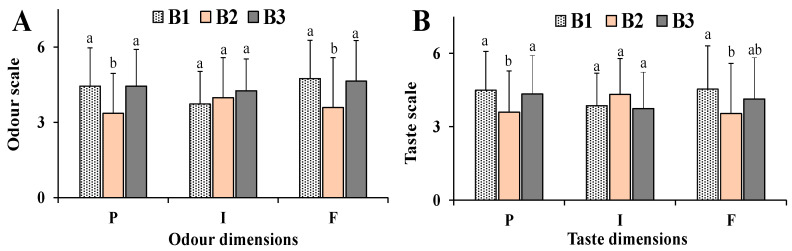
Ratings of pleasantness (P), intensity (I) and familiarity (F) determined for odor (**A**) and taste (**B**) of bottarga sample B1, B2 and B3. Data are presented as mean values ± standard deviations (SD) (*n* = 59). Statistically significant differences between bottarga samples B1, B2, and B3 for each data set were determined by One-way ANOVA followed by the Bonferroni Multiple Comparisons Test. In the same dimension group (P, I and F), mean values sharing the same superscript letters (a,b,c) are not significantly different from each other, while mean values that have no superscript letters in common are significantly different (level of significance: *p* < 0.05).

The evaluation of differences in sensory properties of commercial bottarga samples was then assessed in a human model. Figure 6 shows the ratings of pleasantness, intensity and familiarity determined for the odor and taste of bottarga samples B1, B2 and B3 (59 participants).

B1 and B3 showed the highest values in pleasantness and familiarity for odor quality (in the order B1~B3 > B2) (Figure 6A) and significant statistical differences were observed with respect to B2 sample (*p* < 0.05 for B1 and B3 versus B2 for both dimensions). Most of the participants rated B2 as unpleasant for odor quality. Clear, but not significant, differences were observed in odor intensity among bottarga samples in the following order B3 > B2 > B1. B1 and B3 samples were also quite similar in taste pleasantness (following the order B1≈B3 > B2) (Figure 6B), significantly differing from B2 (*p* < 0.05). B1 showed the highest value in taste familiarity (B1 > B3 > B2) (Figure 6B), significantly differing from B2 sample (*p* < 0.05). Taste intensity of bottarga samples showed a different trend with respect to the odor intensity, with B2 showing the highest value (B2 > B1≈B2). Table 2 shows the correlation coefficients determined among different bottarga odor and taste dimensions. Pleasantness was positively correlated to familiarity for both odor and taste qualities of bottarga samples, as indicated by the correlation coefficient odor pleasantness/familiarity (r = 0.9968) and taste pleasantness/familiarity (r = 0.9672). Moreover, bottarga odor pleasantness and familiarity dimensions were positively correlated with taste pleasantness and familiarity (Table 2), whereas bottarga taste intensity was negatively correlated to pleasantness and familiarity of both odor and taste qualities. The correlations between the total TFA and FFA values of bottarga samples and their odor/taste qualities were determined to understand the potential role of lipid components in the acceptance of this fat-rich food. High negative correlation coefficients were found between bottarga odor intensity/TFA (r = −0.9866) and odor intensity/FFA (r = −0.8297), and similar values were determined with SFA, MUFA, and PUFA (Table 2). Bottarga sample B1, characterized by the highest TFA and FFA amount, was less perceptive by humans for odor intensity than B2 and B3 samples. Moreover, the levels of lipid compounds TFA, SFA, MUFA, PUFA of bottarga samples did not correlate with taste pleasantness, intensity, and familiarity. Some correlations were found between bottarga FFA/taste pleasantness (r = 0.6731) and FFA/taste familiarity (r = 0.8390). Interestingly, a positive correlation was found between bottarga attractant effect to medflies and odor intensity (r = 0.9988) perceived by humans.

**Figure 7:**
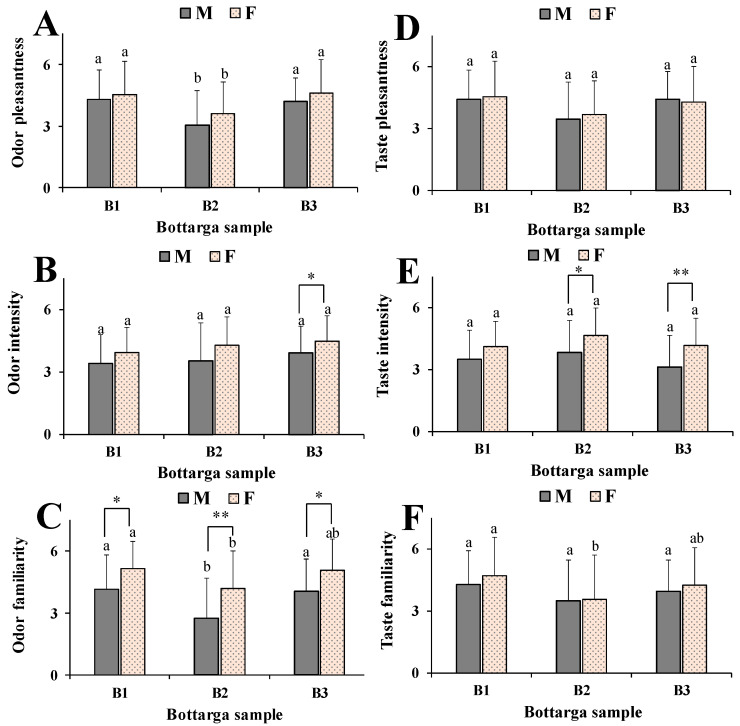
Ratings of odor pleasantness (**A**), intensity (**B**), and familiarity (**C**), and taste pleasantness (**D**), intensity (**E**), and familiarity (**F**) of bottarga samples B1, B2 and B3 in relation to the sex of participants. Data are presented as mean values ± standard deviations (SD) (*n* = 24 for men and *n* = 35 for women). Statistically significant differences between bottarga samples B1, B2, and B3 for each data set in the same sex group (males, M; females, F) were determined by One-way ANOVA followed by the Bonferroni Multiple Comparisons Test. In the same sex group (M and F), mean values sharing the same superscript letters (a, b, c) are not significantly different from each other, while mean values that have no superscript letters in common are significantly different (level of significance: *p* < 0.05). Statistically significant differences between M and F groups for each data set were determined by Student’s unpaired *t*-test with Welch’s correction; ** *p* < 0.01, * *p* < 0.05 for females vs males.

The differences in pleasantness, intensity and familiarity for bottarga odor and taste qualities between women and men were then evaluated in order to evidence the potential role of the gender in the perception of bottarga sensory properties. Figure 7 shows the ratings of pleasantness, intensity, and familiarity of odor (7A–C) and taste (Figure 7D–F) of bottarga samples B1, B2 and B3 in relation to the sex of participants. Men and women showed similar trends in the choice of bottarga samples regarding taste and odor dimensions. However, our data showed that women perceived odor pleasantness, intensity, and familiarity better than men, and a significant better performance in odor familiarity was observed for all bottarga samples (*p* < 0.05 versus men). Women exhibited a greater ability than men to select bottarga samples on the basis of odor intensity (*p* < 0.05 for B3), and a positive correlation was found also between bottarga attractant effect to medflies and odor intensity (r = 0.9902) perceived by women. Women showed a better ability than man in bottarga sample selection on the basis of sensory properties, however, like in the animal model, this ability was negatively correlated to TFA and FFA level/composition, as indicated by the correlation coefficients determined for woman odor intensity/TFA (r = −1.000) and woman odor intensity/FFA (r = −0.9097) (Table 2). As regards taste quality, women exhibited better performance than men only for the intensity of bottarga samples (*p* < 0.01 for B2 and B3).

## 4. Discussion

Bottarga, the salted and semi dried mullet roe, is a rich source of health beneficial *n*-3 PUFA (mainly EPA and DHA) and is recognized as a food product with nutritional and nutraceutical properties [1,2,3,4,5,8,9,10,11,12,13,14,15]. Mullet bottarga, in the Mediterranean countries, is usually added grated to pasta (generally spaghetti) or consumed cut into thin slices together with artichokes, fennel, and extra virgin olive oil. This marine food delicacy has a great traditional and economic value.

We investigated for the first time the differences in the sensory properties (taste and odor) and acceptability of commercial grated bottarga samples in animal and human models in relation to their lipid composition. Visual and microscopic analysis evidenced differences in the color and particle size of the three bottarga samples. B3 showed the most intense color (due to non-enzymatic browning) and the largest size of particles among bottarga samples. The browning process of mullet bottarga is due to complex reactions of reducing sugars and oxidized lipids with amines amino acids, peptides, and proteins (Maillard reaction and protein-lipid interaction) [10,33]. Several factors could affect the color of a commercial grated bottarga sample: the quality of the raw material, the procedures (salting, drying, grinding, and storage) and conditions (time, temperature, and light exposure) of production [6,10,11]. The particle size of grated bottarga samples was correlated to the drying and grinding process. Bottarga samples showed a similar qualitative profile of lipid classes, assessed by HPLC-DAD/ELSD, technique previously used to analyze the composition of lipids extracted from different matrices (mullet bottarga, insects and cells) [13,28]. However, significant differences among bottarga samples were observed in the absolute values of TFA (B1 > B2 > B3) and FFA (B1 > B3≈B2). The FFA level is a consequence of the hydrolytic process induced by the salting and drying procedures on raw materials [5,9]. The traditional production of grated bottarga usually requires a drying process deeper than the preparation of whole bottarga. B1 showed the highest hydrolytic rate, mainly ascribable to a more marked drying process. The differences observed in the TFA absolute values among bottarga samples were due to the different manufacturing procedures that affected the moisture content. The total content of lipids in grated bottarga samples produced in Sardinia was previously estimated in the range of 270–380 mg/g of edible portion [5,10,11,14]. Among the major lipid classes of grated bottarga, a high quantity of WE (approximately 51–73% of total lipids) was found, followed by minor levels of TAG, PL, FC and CE [5,10,13]. Moreover, samples showed a relative high content of FFA (17–29%) and FFAl [5,10]. The extraction of B1, B2 and B3 furnished oils rich in WE and characterized by a high level of FFA/FFAl, exhibiting a lipid profile similar to that of oils previously extracted from different samples of grated bottarga by the chloroforn:methanol mixture [5,10]. Similarly, TC values closely resembled those previously reported for grated bottarga samples (in the range 7.3–10.5 mg/g of edible portion) [5,10,11,14]. Moreover, the FA composition of B1, B2 and B3 lipid extracts was closely similar to that of oils extracted from several commercial grated bottarga samples by chloroforn:methanol published earlier [5,10,11,14]. EPA and DHA in B1, B2 and B3 amounted to 26–28% of total fatty acids, according to literature [5,10,11,14].

Then, a comparative sensory assessment of bottarga samples was performed in insect model (*Ceratitis capitata*). Medflies exhibited the ability to distinguish among bottarga samples (the order of attractiveness was B3 > B2 > B1) and insect females mostly showed this food selection capacity. In our experimental conditions, the insect attractant effect was negatively correlated to the TFA and FFA amount of bottarga samples. Previous insect studies demonstrated that FA could induce contrasted behavioral effects depending on their amount and nature [34]. In *Drosophila melanogaster*, behavioral tests revealed that adults generally preferred SFA compared to UFA. On the contrary, FA alone or combined with volatile substances showed repulsion effects on adult mosquitoes and flies [34]. Our results suggest that high levels of TFA and FFA could be probably less attractant to medflies, however other factors may drive the insect choice among bottarga samples. It is well known that the insect food selection and feeding behavior is initially affected by visual and olfactory information and medfly possess an extremely sensitive and selective olfactory system [35]. The highest attractant effect of B3 sample to medflies could be probably related to its more intense color (due to non-enzymatic browning) than the other samples or maybe medflies showed an olfactory preference for B3 odor due to its higher intensity than B1 and B2. Mullet bottarga is a complex food matrix constitutes by lipids, proteins, water, salt, sugars, vitamins (ascorbic acid, vitamin A), carotenoids and squalene [1,2,5,10]. The flavor chemistry of salted and dried fish roe products is quite complex because browning reaction, lipid oxidation, endogenous enzymes and microbial activity contribute to the development of the final flavor profile [1,10], and salting- and drying-induced proteolysis and lipolysis [10] are important feature for flavor development. Recently, a series of volatile organic flavor compounds, primarily aldehydes and alcohols, was detected in caviar samples as responsible for flavors of fresh fish and seafood, principally deriving from the oxidation of lipid compounds and microbial activity versus amino acids and lipids [36]. Losses of volatile components from a food matrix are usually induced by the process of drying, in relation to several factors (processing temperature, quantity of solids in the food, and vapor pressure of volatile compounds) [37]. In addition, during food product grinding, the temperature rise due to the friction-induced heat during the rupture of particles into smaller dimensions, causes significant losses of flavor components, aroma, and nutrients [38]. Lowest attractant activity of B1 to medflies was probably correlated to its lower odor intensity with respect to other samples, due to its deeper dryness and a more marked loss of aroma due to its lower particles size than B2 and B3. Moreover, the presence in bottarga samples of precursors (i.e., cis-vaccenic acid) of male insect FA-derived pheromones [33] could maybe induce the specific chemosensory/locomotor behavior of female adult medflies. The results of this preliminary study disclosed the insect female ability to select among bottarga samples based on their specific sensory properties, however further studied are needed to clarify the role of bottarga FA and FFA level/composition in the medfly attractiveness.

Finally, the subjective odor and taste perception of bottarga samples was investigated in human. Physiological mechanisms governing the nutritive choice in human are very complex, and biological/psychological variables and social factors may influence food choices [16,17,18]. The perception of food flavor affecting food choice derives from the integration of olfactory/gustatory information and food sensory properties are basically the result of the interaction of food characteristics (chemical/nutritional properties and physical structure) with the characteristics of the consumer (age, gender, physiological/psychological state) and its environment (family, education, and cultural traditions) [17,20]. Volatile organic flavor compounds are responsible for food aroma and are perceived through the smell sensory organs of the nasal cavity [20,39]. Olfactory stimuli activated brain areas such as piriform cortex, orbitofrontal cortex and amygdala and then evoke numerous associations and emotions [23,39]. The olfactory function (smell) is a chemical sense, which has a key role in human life for the detection and evaluation of food taste quality and for avoiding potentially dangerous compounds [40]. The interaction of non-volatile/saliva soluble chemicals with taste receptors on the tongue within the oral cavity determines taste perception [41]. The orbitofrontal cortex provides critical information about food nature, pleasantness, and quality, leading to specific eating responses such as consumption or avoidance [22]. The three bottarga samples evidenced different sensory properties (odor and taste) in the human model. The odor and taste perception of bottarga samples was investigated using a Likert-type scale with 7 points of evaluation [30]. The Likert-type scale is a rating scale [30] used to measure opinions, attitudes, and behaviors of subjects for a specific topic. Similar hedonic scales [42] have been also adopted by the food industry for measuring the acceptability of foods and beverages. B1 and B3 samples emerged as the most preferred samples in terms of odor and taste pleasantness and familiarity. A strong correlation was found among all these dimensions. Unless differences among samples in odor and taste intensity were less marked than other dimensions, B3 revealed the most intense odor and a taste intensity like B1, whereas B2 revealed the most intense and less pleasant taste. Many studies demonstrated a positive correlation between odor familiarity and pleasantness, that represents a consistent result in olfactory research [29]. Moreover, it is well demonstrated that the habitual consumption of a food rises its acceptability [17].

Increasing evidence suggests that humans can detect the fat content of foods through smells and taste, thus perception of fat taste, aroma, and texture is proposed to influence food preferences [23,43,44]. Moreover, the chemoreception of FA seems to implicate different types of lipid sensors [45]. FA may activate the anterior cingulate region, the ventral striatum and the nucleus accumbens which are involved in the satiety paradigm, reward-related learning and in the control of autonomic function [23]. In addition, oral chemosensory detection mechanisms for various types of FFA have been evidenced by behavioral studies [45]. The three bottarga samples showed different olfactory properties, nevertheless, results in the human model did not evidence a clear relation between FA and FFA composition and odor dimensions. Several factors such the quality of the original food matrix, degradative and hydrolytic processes induced by the manufacturing procedures (salting, drying, and grinding) in lipid and protein constituents significantly affect the final flavor of grated mullet bottarga samples [9,10,39,40]. The lowest odor intensity of B1 sample (the richest in TFA and FFA per mg of edible portion) was probably due to the loss of volatile components induced by drying and grinding procedures. However, our results revealed a potential contribution of FFA in the pleasantness and familiarity dimensions of taste, but not in taste intensity, confirming the potential involvement of FFA in fatty food recognition [24,26], with important implications in food choice, acceptance, and consumption. Due to the complex composition of bottarga, several macronutrients (fat protein and carbohydrates) and sodium may affect the total taste response in the human model [45,46]. Most of these nutrients have specific sensory properties and may be pleasant or unpleasant for the consumer. Therefore, in complex food matrices, it is difficult to provide evidence of clear relationships between the concentration of individual chemical stimuli and consumer physiological perception and reaction [45,46]. Bottarga sample B1, characterized by the highest TFA and FFA amount, was less perceptive by humans for odor intensity than B2 and B3 samples like in the insect model. The positive correlation found between bottarga attractant effect on medflies and odor intensity perceived by humans, confirmed that the odor intensity of bottarga samples could be probably the most important factor in the selective attractant effect on medflies. Insects and humans exhibited similar trend in the selection of bottarga samples based on olfactory perception, however insect selection ability was more marked than humans.

Interestingly, significant differences were observed in pleasantness, intensity and familiarity for bottarga odor and taste qualities between women and men, shedding light on the potential role of the gender in the perception of bottarga sensory properties. Women, like females in the insect model, exhibited a greater ability than men to select bottarga samples based on their better olfactory perception. A previous study [47] reported that females exhibited better olfactory ability compared to males. The reason of this better olfactory perception in women is related to a complex interaction between the olfactory system and gonadal hormones and in women the olfactory perception is associated to the fluctuation of menstrual cycle [48].

## 5. Conclusions

Our study for the first-time investigated differences in the sensory properties (taste and odor) and acceptance of grated mullet bottarga samples, a natural source of health-beneficial *n*-3 PUFA, in relation to their lipid composition. Bottarga samples were characterized by differences in the amounts of TFA and FFA and showed different sensory properties in insects and human models. Taken together, our data evidenced a potential contribution of the FFA amount in the pleasantness and familiarity dimensions of bottarga sample taste (but not in taste intensity and odor dimensions), suggesting the FFA potential role in the choice and acceptability of this traditional marine fat-rich food. Insect female and women exhibited the best ability to select among bottarga samples based on their sensory properties. Bottarga is a complex food matrix and several macronutrients like salt, fats and proteins and their degradative and hydrolytic products could affect its resultant odor and taste qualities, making very hard to evaluate the real contribution of TFA and FFA in the oro-sensory properties and consumer acceptance of bottarga. The results on the bottarga sensory properties described within this research represent a significant contribution to the existing literature, useful to increase the knowledge about this valuable marine food product with nutritional and nutraceutical properties.

## Figures and Tables

**Table 1:** Fatty acid (FA) composition (expressed as %, g/100 g of total fatty acids) of fixed oils obtained from grated bottarga samples (B1, B2 and B3).

Common Name	CA:DB ^1^	B1	B2	B3
Lauric acid	12:0	trace	trace	trace
Myristic acid	14:0	3.02 ± 0.16	3.14 ± 0.21	3.22 ± 0.66
Palmitic acid	16:0	11.78 ± 0.64	10.60 ± 0.94	10.07 ± 0.43
Palmitoleic acid	16:1 *n*-7	16.51 ± 0.46	14.15 ± 0.26	13.81 ± 0.24
Hexadecadienoic acid	16:2	1.29 ± 0.06	1.59 ± 0.12	1.52 ± 0.10
Hexadecatrienoic acid	16:3	0.65 ± 0.01	0.84 ± 0.06	0.74 ± 0.01
Hexadecatetranoic acid	16:4	0.10 ± 0.00	0.32 ± 0.02	0.24 ± 0.01
Stearic acid	18:0	4.24 ± 0.58	5.50 ± 0.73	5.80 ± 0.08
Oleic acid	18:1 *n*-9	10.34 ± 0.46	13.82 ± 0.38	14.34 ± 0.09
cis-Vaccenic acid	18:1 *n*-7	6.38 ± 0.21	5.69 ± 0.24	5.05 ± 0.17
Linoleic acid	18:2 *n*-6	4.68 ± 0.20	4.77 ± 0.70	5.61 ± 0.34
γ-Linolenic acid	18:3 *n*-6	0.97 ± 0.07	0.44 ± 0.05	0.40 ± 0.03
α -Linolenic acid	18:3 *n*-3	1.29 ± 0.04	0.93 ± 0.02	0.97 ± 0.04
Stearidonic acid	18:4 *n*-3	1.66 ± 0.02	1.72 ± 0.07	1.75 ± 0.04
Eicosatrienoic acid	20:3 *n*-6	2.94 ± 0.19	2.42 ± 0.06	2.51 ± 0.11
Arachidonic acid	20:4 *n*-6	2.74 ± 0.07	0.85 ± 0.01	0.85 ± 0.03
Eicosapentaenoic acid	20:5 *n*-3	10.51 ± 0.43	12.98 ± 0.58	12.82 ± 0.41
Docosapentaenoic acid	22:5 *n*-3	5.06 ± 0.14	5.06 ± 0.25	4.57 ± 0.18
Docosahexaenoic acid	22:6 *n*-3	15.86 ± 0.77	15.18 ± 0.43	15.73 ± 0.40
Saturated fatty acids	SFA	19.03 ± 1.06	19.24 ± 0.31	19.09 ± 0.32
Monounsaturated fatty acids	MUFA	33.23 ± 0.90	33.66 ± 0.51	33.20 ± 0.45
Polyunsaturated fatty acids	PUFA	47.74 ± 0.96	47.09 ± 0.37	47.71 ± 0.30

^1^ Abbreviations: Carbon Atoms:Double Bonds (CA:DB). Bottarga oil analysis was performed in quadruplicate and all data are expressed as mean values ± standard deviations (SD); (*n* = 4).

**Table 2:** Correlation coefficients (r) calculated between sets of data determined for bottarga samples B1, B2 and B3.

Data	FFA	IA	OP	OI	OF	TP	TI	TF
TFA	0.9075	−0.9909	0.1451	−0.9866	0.2234	0.3002	0.0506	0.5328
SFA	0.8550	−0.9996	0.0397	−0.9983	0.1192	0.1977	0.1559	0.4404
MUFA	0.8466	−1.0000	0.0178	−0.9993	0.0974	0.1762	0.1775	0.4206
PUFA	0.9528	−0.9663	0.2673	−0.9585	0.3432	0.4168	−0.0743	0.6342
FFA	1	−0.8426	0.5472	−0.8267	0.6122	0.6731	−0.3736	0.8390
IA	−0.8426	1	−0.0104	0.9996	−0.0900	−0.1689	−0.1848	−0.4139
OP	0.5472	−0.0104	1	0.0186	0.9968	0.9873	−0.9808	0.9146
OI	−0.8267	−0.9996	0.0186	1	0.2234	−0.1403	−0.2132	−0.3873
OF	0.6122	−0.0900	0.9968	0.2234	1	0.9968	−0.9622	0.9439
TP	0.6731	−0.1689	0.9873	−0.1403	0.9968	1	−0.9375	0.9672
TI	−0.3736	−0.1848	−0.9808	−0.2132	−0.9622	−0.9375	1	−0.8182
TF	0.8390	−0.4139	0.9146	−0.3873	0.9439	0.9672	−0.8182	1

Abbreviation: TFA, total fatty acids; SFA, saturated fatty acids; MUFA, monounsaturated fatty acids; PUFA, polyunsaturated fatty acids; FFA, free fatty acids; IA, insect attractiveness; OP, odor pleasantness; OI, odor intensity; OF, odor familiarity; TP, taste pleasantness; TI, taste intensity; TF, taste familiarity.
